# Rescaling Flow Curves of Protein-Stabilized Emulsions

**DOI:** 10.3390/nano15090650

**Published:** 2025-04-25

**Authors:** Santiago F. Velandia, Philippe Marchal, Véronique Sadtler, Cécile Lemaitre, Daniel Bonn, Thibault Roques-Carmes

**Affiliations:** 1 Université de Lorraine, CNRS, LRGP, F-54000 Nancy, France; sf.velandia10@gmail.com (S.F.V.); philippe.marchal@univ-lorraine.fr (P.M.); veronique.sadtler@univ-lorraine.fr (V.S.); cecile.lemaitre@univ-lorraine.fr (C.L.); 2 Van der Waals-Zeeman Institute, Institute of Physics, University of Amsterdam, Science Park 904, 1098 XH Amsterdam, The Netherlands; d.bonn@uva.nl

**Keywords:** rheological modelling, jamming transition, scaling of flow curves, viscoelastic behavior, Cox–Merz rule

## Abstract

In this study, we investigate the flow behavior of oil-in-water Pickering emulsions stabilized with bovine serum albumin (BSA). Through the use of a phase transition analogy and scaling parameters previously applied to surfactant-stabilized emulsions, we successfully describe the flow behavior, suggesting remarkable similarity in the rheology of these emulsion categories. Additionally, we explore the possibility of extending this modeling framework to the oscillatory mode. Above the jamming fraction, the scaled data in the oscillatory regime present a similar trend as the rotational rheology curves. However, upon closer examination of the scaling conditions, it becomes evident that the rescaling does not accurately describe the behavior of $\left|G^{*}\right|$. Despite this, our findings shed light on the universality of scaling parameters and provide valuable insights into the rheological behavior of these complex fluids.

## 1. Introduction

**Figure 1:**
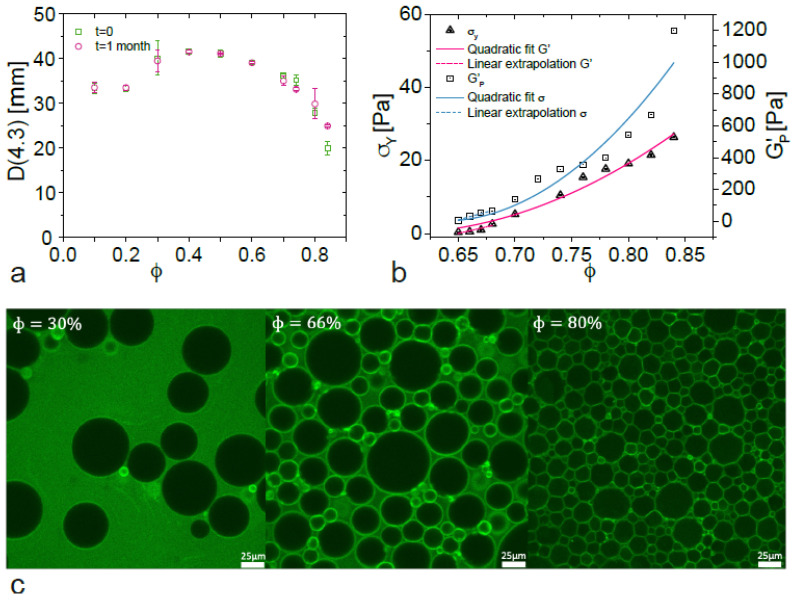
(**a**) Average droplet size variation (D(4.3)) as a function of ϕ and time for silicone oil-in-water emulsions stabilized with 1 wt% BSA at pH 5. Evolution through 1 month of the average droplet size (D(4.3)). “t = 0” represents the average droplet size just after preparation. “t = 1 month” corresponds to the average droplet size after 1 month of storage. (**b**) Critical volume fraction determination for silicone oil-in-water emulsions stabilized with BSA particles. The yield stress and the elastic modulus plateau values as a function of the dispersed-phase volume fraction are fitted to quadratic and linear regression laws of the form: $\sigma_{y}=\alpha {\;\left(\phi -\phi_{c}\right)}^{2}$, ${G\prime }_{p}=\alpha {\;\left(\phi -\phi_{c}\right)}^{2}$, $\sigma_{y}=g\;\phi +d$, or ${G\prime }_{p}=g\;\phi +d$ (with α, $\phi_{c}$, g and d fitting parameters) to determine ϕ_C_. (**c**) Fluorescence intensity images of fluorescein isothiocyanate (FITC)–BSA conjugate in BSA-stabilized emulsions with varying oil fractions. The scale represents 25 µm.

Complex viscoelastic behavior arises in jamming systems due to their multi-component structure. These fluids are present in our daily lives as emulsions, foams or pastes and understanding their rheological behavior represents a fundamental and industrial interest. From a rheological point of view, the main feature of jamming systems is the existence of a critical volume fraction (ϕ_C_), at which a change from a fluid-like state to a solid-like state is observed [1,2,3,4]. As an illustration, the transition in microscopic structure for an oil-in-water emulsion from spherical droplets to hexagonal packing before and after exceeding ϕ_C_ is shown in Figure 1 inset. Conventionally, surfactants are used to stabilize such systems by reducing interfacial tension. The search for less ecologically harmful emulsifiers is currently receiving a lot of attention. Pickering emulsions, also known as particle-stabilized emulsions, are a good substitute [5]. In the recent years, multiple systems of Pickering emulsions with varying particle nature have been reported [6,7,8]. Using particles instead of surfactants represents a great opportunity to design and obtain innovative environmentally friendly products. For instance, globular proteins as Pickering stabilizers are a great example of an alternative to classical surfactants [9,10]. Due to their stability and the globular structure of proteins such as albumin, these objects are considered as soft particles [11]. Although, in principle, these macromolecules can have an amphiphilic character and decrease the interfacial tension like classical surfactants, multiple investigations also report a Pickering stabilization mechanism [12,13,14]. The stabilization mechanism of Pickering systems is therefore still a matter of debate [8,15,16]. Pickering emulsions are considered to exhibit a high resistance to coalescence because of the large amount of energy required to desorb a particle from the interface [17]. Nevertheless, it has been observed that at low strain rates it is possible to dislodge particles from interfaces of Pickering emulsions [8]. Thus, it has also been considered that the stabilization mechanism is mainly based on a steric barrier of particles around the emulsion droplets. In addition, free particles in the bulk have been said to play a key role in increasing the steric protection against coalescence [15,18,19,20,21]. Specifically, for protein-stabilized emulsions, regardless from the mechanism, it has been shown that these macromolecules approach the oil phase from their hydrophobic regions, adsorb on the oil–water interfaces, reorganize by changing conformation and build an interfacial film providing protection against coalescence [11]. It has also been shown that proteins produce a coupled stabilization mechanism combining a steric barrier from Pickering adsorption and molecular adsorption decreasing interfacial tension as surfactants [22]. Nevertheless, this is still subject to discussion and demonstrates how challenging it is to analyze and predict their rheological behavior.

Since a critical volume fraction is the primary rheological signature of fluids that exhibit a jamming transition, rheological behavior modelling frequently begins with taking this property into account. The critical volume fraction (ϕ_C_) is a function of multiple parameters such as the complex fluid nature, the system polydispersity and the interdroplet interactions [23]. A reference value is ϕ_RCP_ = 0.641 corresponding to the random close packing of monodisperse spheres [24]. Above ϕ_C_, the flow behavior is predominantly elastic under low deformations and a critical shear stress, called the yield stress, is required to start flowing. This is often described by the Herschel–Bulkley model [25]:

(1) $$\sigma =\sigma_{Y}+K\;{\dot{\gamma }}^{\beta }$$

where σ_y_ is the yield stress, $\dot{\gamma }$ is the shear rate, while K and β are adjustable parameters also referred to as the fluid consistency and the flow index parameters, respectively [26]. In the same manner, when ϕ < ϕ_C_ the flow behavior is mainly Newtonian under low shear rates and presents shear-thinning behavior at higher shear rates. This can be successfully described by the Cross model [27]:

(2) $$\sigma =\eta_{1}\dot{\gamma }/(1+C\;{\dot{\gamma }}^{1-\delta })$$

In this case, $\eta_{1}$ is the Newtonian plateau viscosity, $\dot{\gamma }$ is the shear rate, while C and δ are adjustable parameters. The effect of the volume fraction on the Newtonian plateau $\eta_{1}$ is commonly described as a power law of ϕ and ϕ_C_, as in the case of the Krieger–Dougherty model for the viscosity [28,29]:

(3) $${\eta_{1}=\eta_{s}\left(1-\frac{\Phi }{\Phi_{c}}\right)}^{-m}$$

where $\eta_{s}$ is the solvent viscosity, m is an adjustable parameter and η_1_ is the low-shear viscosity also involved in Equation (2). It has also been shown that similar power law-based equations can be applied to describe σ_y_ behavior as well as other important rheological characteristics such as the shear modulus. Dinkgreve et al. considered Quemada-type equations to describe both properties so that [30]:

(4) $$\sigma_{Y}=\sigma_{0}{\left|\Delta \phi \right|}^{\Delta },\;G=G_{0}{\left|\Delta \phi \right|}^{B}$$

where $\sigma_{0}$, $G_{0}$, Δ and B are adjustable parameters. Both properties can also be related to the yield strain (γ_y_) in the solid-like state of the system by σ_y_ = G γ_y_. The exponents Δ and B from Equation (4) have shown to follow Δ ≈ B ≈ 2 in experiment and simulation studies which also implies that γ_y_ is roughly constant [30,31,32]. It is worth mentioning that other authors such as Paruta-Tuarez et al. or Princen and Kiss have introduced similar models before to describe the modulus in the linear viscoelastic region, following similar expressions as in Equation (4) (see Supplementary Materials for more details) [15,28,33,34,35].

The previously mentioned models are able to correctly describe the flow or oscillatory rheological behavior. However, these parameters are difficult to associate with the fundamental behavior of the complex fluids being evaluated. In response to this, another approach has been developed in recent years to model the rheological behavior while bringing more fundamental insight. It has been shown that the fluid to solid passage in flow behavior can be treated like a phase transition phenomenon [24]. Paredes et al. experimentally demonstrated that flow curves of complex systems can be re-expressed as a function of ϕ_C_ and two parameters by scaling the shear rate and shear stress as: ${\dot{\gamma }}_{scaling}=\dot{\gamma }/{\left|\Delta \phi \right|}^{\Gamma }$ and $\sigma_{scaling}=\sigma /{\left|\Delta \phi \right|}^{\Delta }$ with $\Delta \phi =\left|\phi -\phi_{c}\right|$ [36]. This procedure allows to collapse the ϕ > ϕ_C_ data into a master curve and automatically collapse the ϕ < ϕ_C_ curves into a secondary master curve. Interestingly, both master curves are only described by ϕ_C_ and the two scaling parameters Δ and Γ. The master curves show the same behavior of any jamming system below and above ϕ_C_ so that Equations (1) and (2) can be used to evaluate the scaling procedure. Furthermore, these models can now be linked to the scaling approach such that for supercritical behavior β_Herschel–Bulkley_ = Δ/Γ. The subcritical behavior can also be related to the scaling procedure through Equation (3) considering: m_Krieger_ = Γ − Δ. According to the phase transition analogy, Paredes et al. considered that Δ and Γ had a universal character. From that, they successfully applied it to a model emulsion system stabilized with anionic surfactants obtaining Δ and Γ close to 2 and 4, respectively [36]. Subsequently, Dinkgreve et al. tested the scaling approach by experimentally evaluating flow curves of emulsions with mobile droplet surfaces (harmonic interactions between droplet interfaces) and emulsions with rigid droplet surfaces [30]. Some differences in the scaling parameters were expected due to the variation in the mechanical properties of each system. Their experimental results demonstrated the validity of the scaling methodology by correctly modelling the flow behavior of each system. Surprisingly, as for Paredes et al., the scaling parameters remained in the vicinity of 2 and 4 for Δ and Γ for both cases regardless of the differences in the mechanics of the emulsion interfaces. This provided further support for the hypothesis about the universality of the scaling parameters.

Moreover, Dinkgreve et al. successfully applied the scaling approach to athermal emulsions and thermal systems (emulsions and nanoparticle suspensions) [4]. The authors extended the scaling procedure by considering a stress scale σ_0_ for athermal and thermal fluids. The new scaling procedure followed: ${\dot{\gamma }}_{scaling}=\dot{\gamma }\;\eta_{0}/\sigma_{0}{\left|\Delta \phi \right|}^{\Gamma }$ and $\sigma_{scaling}=\sigma /\sigma_{0}{\left|{\Delta \phi }_{J}\right|}^{\Delta }$ with η_0_ in the order of the solvent viscosity and σ_0_ corresponding to the stress scale. For the athermal case, σ_0_ is given by the Laplace pressure Σ with the interfacial tension γ and the average droplet size R: Σ = γ/R. In the case of the thermal system, σ_0_ is obtained by the osmotic pressure given by (k_B_ T)/R^3^. This allowed to rescale both type of fluids onto the same master curves while keeping the scaling parameters close to the same values mentioned before. However, the question of universality remains open since more complex fluids (e.g., emulsions stabilized with particles that we consider here) must be evaluated before the universal nature of the scaling parameters can be asserted.

In addition to rotational rheology, we will here also consider the behavior in oscillatory rheology. At a fundamental and practical level, oscillatory rheology provides valuable information about the system stability, the yield conditions and the extent of inter-particle interactions [15,32]. Access to describe and predict structural information in the same way as for rotational rheology with the scaling procedure could have great impact to design and handle complex fluids. However, little has been performed on this issue. Tighe combined a theoretical approach and simulations to predict a collapse of G*(ω) by determining the distribution of relaxation rates on a model packed system [31]. Such results showed a collapse of the elastic and loss moduli by means of different scaling parameters than those from flow curves mentioned before. On the other hand, Dagois-Bohy et al. reported the alternative to scale oscillatory data as a function of the confining pressure instead of the distance to jamming [37]. Interestingly, a collapse of G′ and G″ was also observed for simulations as a function of confining pressure. It is worth noting that these results were obtained under experimentally inaccessible measurement conditions with a constant strain value of γ_0_ = 10^−10^. While the scaling procedure follows a similar methodology than the work of Dinkgreve et al., the resulting scaling parameters differed considerably [4]. Dekker et al. sought to close the gap between these broad differences by numerically relating the volume fraction with the confining pressure which allowed to identify that different windows of response were being scaled due to the limits of experimental conditions compared to simulations [38].

In this paper, we seek to explore the scaling procedure in model protein-stabilized emulsions. For this, we use silicone oil-in-water emulsions stabilized with bovine serum albumin (BSA). In a first instance, the scaling of the flow behavior of protein-stabilized emulsions is investigated. To our knowledge, this is the first time that this modelling method is applied to jammed emulsions stabilized with soft particles of this nature. Additionally, by applying the scaling framework to the complex shear modulus behavior, we investigate the possible relationship between rotational and oscillatory behavior. It is important to confirm if the scaling parameters obtained with surfactants and particles stabilizers remain similar to those obtained here with soft proteins, leading to a kind of universal behavior regardless of the nature of the emulsifier. In the same time, this study aims to go further in the debate about the behavior of proteins as emulsion stabilizers which can behave as surfactants and/or particles. In addition, this is the first time that the rheological behavior of proteins is studied in such fine details.

## 2. Materials and Methods

### 2.1. Materials

Silicone oil (0.96 g/mL density at 25 °C, 50 cSt kinematic viscosity at 25 °C) and Fraction V bovine serum albumin (BSA) were purchased from Sigma-Aldrich FR (Paris, France). Anhydrous sodium acetate (ReagentPlus^®^, ≥99%), glacial acetic acid (ReagentPlus^®^, 99%), sodium azide (ReagentPlus^®^ ≥99.5%), hydrochloric acid (HCl) and all other reagents were used as received unless specified. Fluorescein isothiocyanate (FITC)–BSA conjugate was obtained from Sigma Aldrich (Paris, France).

### 2.2. Preparation of BSA Solutions

Milli-Q filtrated and deionized water (18 M.cm resistivity) was used to prepare an acetate buffer solution. As these proteins have shown higher interfacial tension decrease under electrically neutral conditions, the pH of the buffer solution was set to 5, which corresponds to the interval for isoelectric conditions of BSA (between 4.8 and 5.6) [39]. Then, a 1 wt% BSA solution was prepared by a simple process of magnetic stirring for 4 h. Note that a low-speed magnetic stirring process was used to prevent foaming of the BSA solutions. In details, the volume of 100 mL of solution inside a beaker of 250 mL was stirred with a magnetic stirrer of a dimension of 2–3 cm at a speed range of 300–500 rpm. Throughout all the experiments, the ionic strength remained constant and determined by the buffer concentration at 5 mM. Subsequently, 0.02 wt% of sodium azide (antimicrobial) was added and the resulting solution was stored at 4 °C overnight before use. The maximum allowable shelf life for using the solutions was 5 days. The BSA hydrodynamic radius was 5 nm with a polydispersity index of 0.137, obtained through dynamic light scattering measurements (DLS). For this purpose, the DLS analysis on BSA solutions was conducted by the means of a ALV LSE-5003 goniometer coupled to a JDSU 1145P laser with a wavelength of 633 nm. Similar particle sizes have been reported elsewhere for BSA proteins in solution [10,13].

### 2.3. Emulsion Preparation

Silicone oil-in-water emulsions were prepared with a variable dispersed-phase volume fraction from 0.1 to 0.84. The total volume of the emulsions was kept constant at 20 mL. The oil phase was added instantly to a 1 wt% BSA solution and emulsified with an Ultra-Turrax turbine blender (IKA T25 Basic/Dispersion Tool S25-NK-19G, Sigma-Aldrich, France) at a rate of 21,000 rpm for 1 min at 20 °C. Throughout the emulsification, the Ultra-Turrax head was moved from bottom to top to prevent local dead zones during agitation. The temperature of the emulsion was maintained constant during the stirring process using a cooling thermostat bath. The temperature was fixed to 20 °C. During the preliminary experiments, care was taken to ensure that the temperature remained constant (at 20 °C) during the emulsion preparation thanks to the cooling thermostat bath. To this aim, the temperature of the emulsion was followed during the 1 min of agitation with the Ultra-Turrax turbine blender. The results indicated that the temperature remained in the range of 20–25 °C and never went above 25 °C. Consequently, the temperature remained far from a temperature of 50 °C, which is the temperature of thermal denaturation of BSA. Considering the short stirring time required for emulsification as well as the numerous studies indicating that thermal denaturation of BSA starts above 50 °C, it is assumed that there is no denaturation of proteins by mechanical agitation [40,41]. The samples were characterized immediately after emulsification and were stored under refrigerated conditions (4 °C). Dilution tests with acetate buffer solutions were performed allowing to verify the absence of phase inversion as well as the oil-in-water nature of the prepared emulsions.

### 2.4. Droplet Size Determination

The average droplet size in the emulsion was determined from static light measurements by means of a Mastersizer 3000 granulometer (Malvern^®^, Worcestershire, UK). A refractive index of 1.405 was used for silicone oil. In the studied range of oil fractions, a monomodal distribution was observed with varying size. To identify a trend in droplet size variation, measurements were performed above and below the critical oil fraction. All measurements were performed by triplicate.

Confocal laser scanning microscopy (CLSM) was also used to evaluate the microscopic structure of the emulsions stabilized with BSA. For this purpose, the confocal microscope TCS-SP8 (Leica, Mannheim, Germany) was used. The apparatus was equipped with a hybrid detector. BSA solution containing 1 μM of FITC–BSA conjugate displayed an absorption maximum at 492 nm and an emission maximum at 520 nm. Consequently, the wavelength of the laser was fixed at 470 nm for the measurements. The emission range was fixed between 500 nm and 700 nm. In terms of droplets «development», it is important to keep in mind that the oil and, consequently, the droplets were not directly marked with any fluorescent dye. However, the droplets were observed through the surrounding proteins and they were indeed equivalent (in size) to what was observed in particle size distribution analysis. This last measurement was made with the refractive index of the oil and this indicated that there were indeed droplets of silicone oil in the images.

### 2.5. Rheological Measurements

The rheological behavior of BSA-stabilized emulsions was studied with a controlled shear stress (CSS) AR-G2 rheometer from TA instruments (Paris, France). A MCR 302 rheometer from Anton Paar was also used to measure the oscillatory behavior. In the first case, a 40 mm diameter parallel plate geometry was used to perform oscillatory and rotational tests. The gap and working temperature were kept constant at 1 mm and 20 °C, respectively. In a second instance, a 50 mm-diameter cone-and-plate geometry (1° cone) with roughened surfaces to prevent wall slip was used. All samples were pre-sheared at 100 s^−1^ for 30 s followed by a 30 s rest period to ensure the same initial conditions. The oscillatory and rotational tests were carried out separately and by changing the sample used. Rotational tests consisted of shear rate sweeps from 10^3^ s^−1^ to 10^−3^ s^−1^. Oscillatory strain sweeps from 10^−3^ to 10^2^ amplitudes were performed at a fixed frequency of 1 rad/s to identify the linear viscoelastic region. From that, oscillatory frequency sweeps were performed from 10^2^ rad/s to 10^−1^ rad/s at a constant strain of 1% for concentrated emulsions and 10% for diluted conditions. Shear elastic (G′), viscous (G″), complex modulus $\left|G^{*}\right|$ and complex viscosity η* were recorded to study the viscoelasticity of emulsions. $\left|G^{*}\right|$ accounts for the viscoelastic behavior of our systems given that it depends both on G′ and G″ modulus as $G^{*}=\sqrt{{G^{\prime }}^{2}+{G^{\prime \prime }}^{2}}$. As a general feature, the samples did not present thixotropy when performing verification experiments by concatenating shear rate sweeps (see Supplementary Materials). For greater clarity, only a few curves in the subcritical region were taken into account for frequency sweeps.

Every experiment was repeated at least three times. For each dispersed-phase fraction tested, 3 separate emulsions were formulated. Each rheological analysis was performed 3 times with the 3 different samples so that each curve reported on the different graphs is the result of the average of 3 different rheograms. Considering two mean viscosity values ${\bar{\eta }}_{1}$ and ${\bar{\eta }}_{2}$ obtained from N_1_ and N_2_ repetitions on emulsions with volume fractions ϕ_1_ and ϕ_2_, the difference between ${\bar{\eta }}_{1}$ and ${\bar{\eta }}_{2}$ will be considered statistically significant if:

(5) $$R=\frac{{\bar{\eta }}_{1}-{\bar{\eta }}_{2}}{\sqrt{\frac{s_{1}^{2}}{N_{1}}+\frac{s_{2}^{2}}{N_{2}}}}>N\;(0,1)(p)$$

where N (0,1)(p) is the value of the reduced centered normal variable for the chosen safety threshold p (*p*-value); $s_{1}^{2}{/N}_{1}$ and $s_{2}^{2}{/N}_{2}$ are the variances of ${\bar{\eta }}_{1}$ and ${\bar{\eta }}_{2}$; ${(s}_{1}^{2}{/N}_{1}+s_{2}^{2}{/N}_{2})$ is the variance of ${(\bar{\eta }}_{1}-{\bar{\eta }}_{2})$. In this case, N(0,1)(0.05) = 2 with the *p*-value set at 0.05. Each rheogram being repeated three times, N_1_ = N_2_ = 3. Furthermore, rheological measurements typically have a relative accuracy of 5%. This value takes into account the calibration of the torquemeter, the calibration of the optical tachometer, the temperature control of the samples, the exactness of the gap, the geometrical precision of the measurement tools (parallel plates in this case) and the measurement repeatability. Under these conditions, for each viscosity value, $s_{i}=0{.05\;\eta }_{i}$, leading to:

(6) $$R=\frac{{\bar{\eta }}_{1}-{\bar{\eta }}_{2}}{\sqrt{\frac{s_{1}^{2}}{N_{1}}+\frac{s_{2}^{2}}{N_{2}}}}=\frac{{\bar{\eta }}_{1}-{\bar{\eta }}_{2}}{\sqrt{\frac{(0{.05\;\bar{\eta }}_{1})2}{3}+\frac{(0{.05\;\bar{\eta }}_{2})2}{3}}}=\frac{\sqrt{3}}{0.05}\frac{{\bar{\eta }}_{1}-{\bar{\eta }}_{2}}{\sqrt{{\bar{\eta }}_{1}^{2}+{\bar{\eta }}_{2}^{2}}}$$

**Figure 2:**
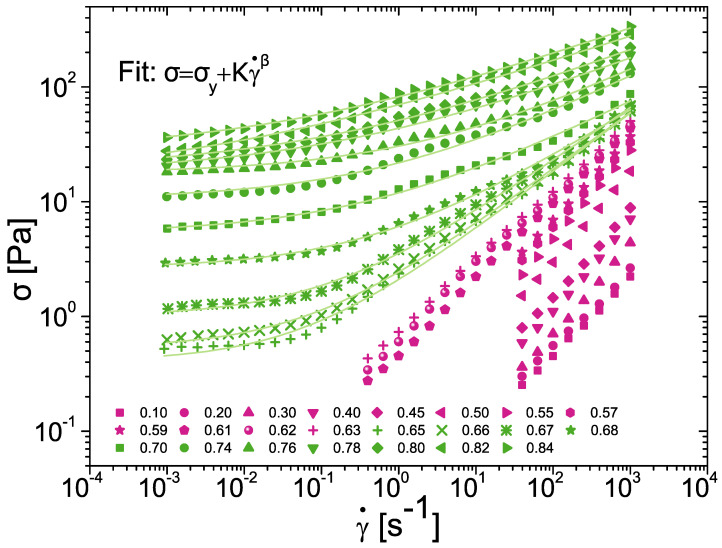
Flow curves of silicone oil-in-water emulsions stabilized with 1 wt% BSA and varying dispersed-phase volume fractions from 0.1 to 0.84. The supercritical (green) curves are fitted to the Herschel–Bulkley model (green lines).

**Figure 3:**
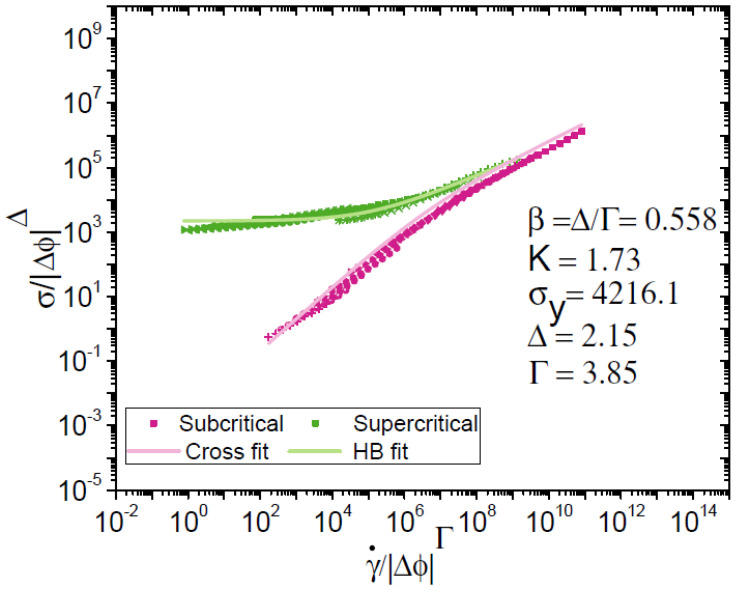
Master flow curves showing the supercritical (ϕ > ϕ_C_) and subcritical (ϕ < ϕ_C_) branches of collapsed flow curves as a function of the distance to ϕ_C_.

**Figure 4:**
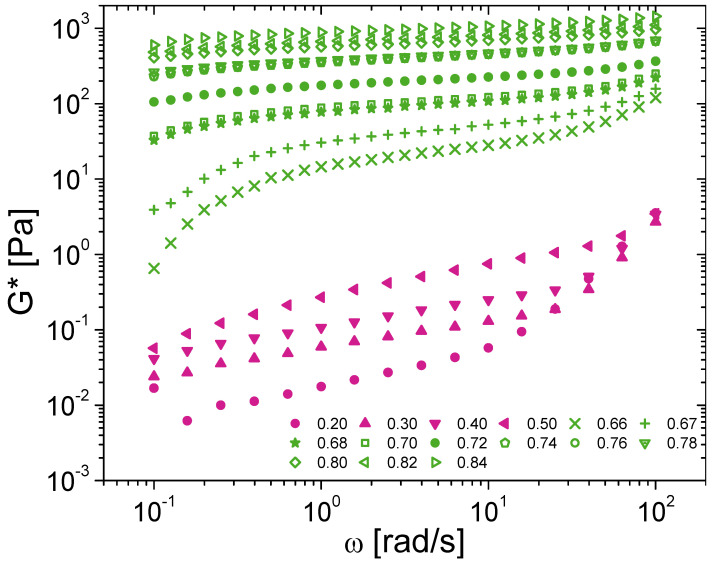
Frequency sweeps of silicone oil-in-water emulsions with 1 wt% BSA and varying dispersed-phase volume fraction in the subcritical (purple) and supercritical (green) regions from ϕ = 0.20 to ϕ = 0.84.

**Figure 5:**
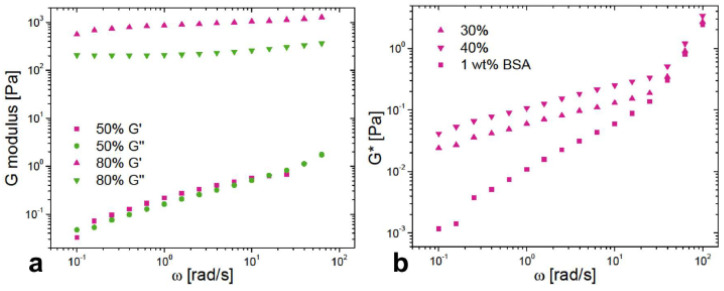
Frequency sweeps for different systems containing BSA. (**a**) Storage and loss moduli for emulsions stabilized with BSA below and after the critical jamming fraction. Samples shown here are prepared with 1 wt% BSA and varying oil fractions (ϕ = 0.50 (“50%”) or ϕ = 0.80 (“80%”)) (**b**) Comparison between rheological behavior of a BSA solution at 1 wt% and diluted emulsions (ϕ = 0.30 (“30%”) or ϕ = 0.40 (“40%”)).

**Figure 6:**
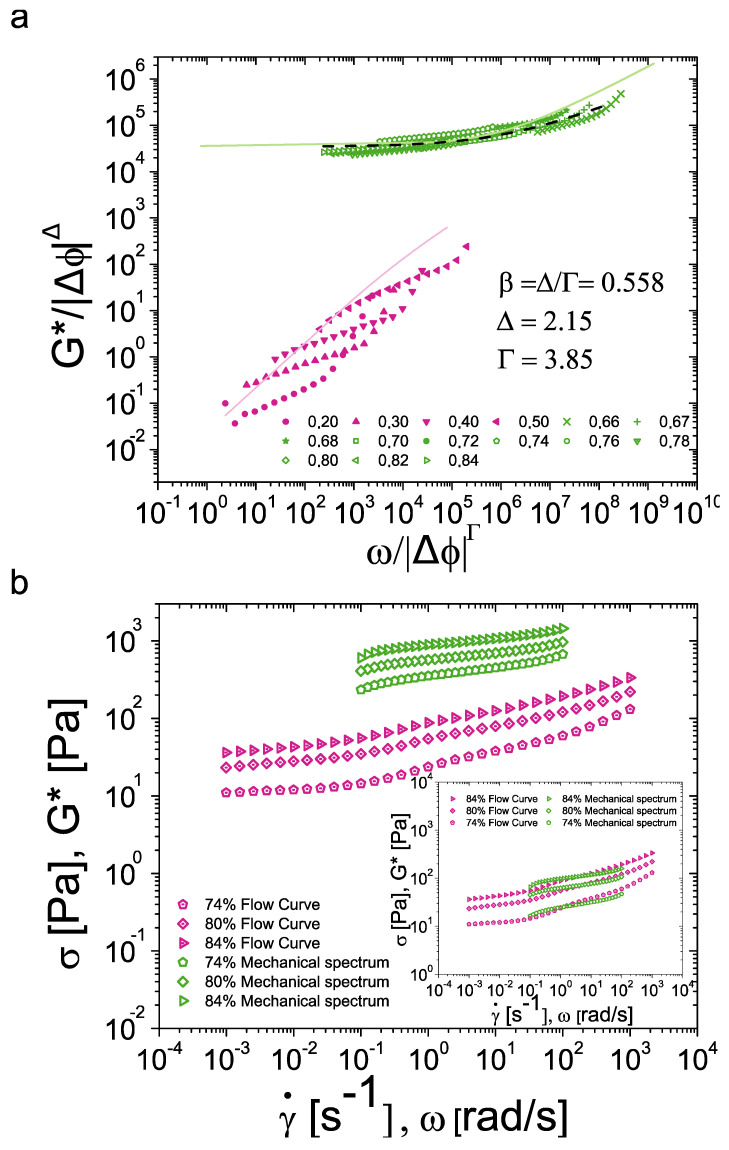
Connecting oscillatory to flow behavior. (**a**) Scaling of oscillatory data showing the supercritical (ϕ > ϕ_C_) branch of collapsed frequency sweeps (green) and the subcritical curves (purple) as a function of the distance to ϕ_C_. The green line corresponds to the fit of the Herschel–Bulkley model, taken from the scaling of flow curves. The doted-lines show the equivalent Herschel–Bulkley model applied to collapsed data, while the pink line correspond to the modified Cross equation also taken from the scaling of flow curves. (**b**) Cox–Merz rule applied to: shear stress (“Flow Curve”) and $\left|G^{*}\right|$ modulus (“Mechanical spectrum”) of silicone oil-in-water emulsions with ϕ > ϕ_C_ and 1 wt% BSA. Inset: the ratio between $\left|G^{*}\right|$ and σ is relatively constant and $\left|G^{*}\right|$ data can be collapsed with a constant yield strain γ_y_.

**Figure 7:**
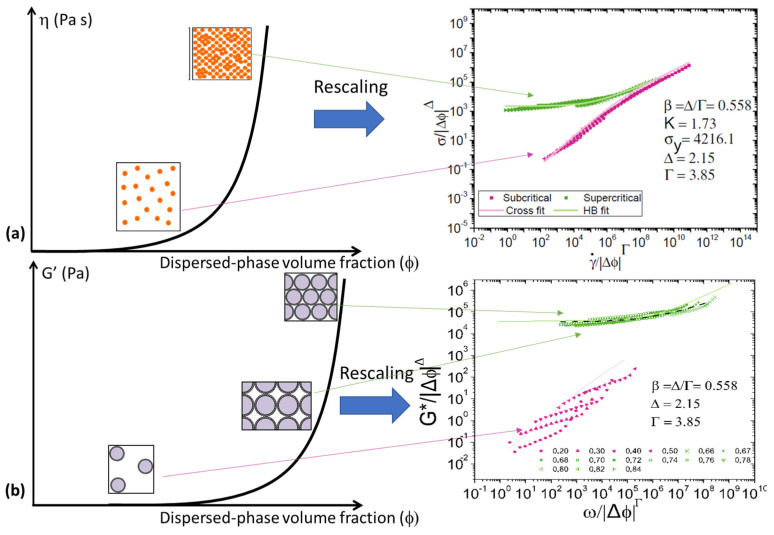
Graphical models summarizing the rescaling approach: (**a**) flow behavior and (**b**) oscillatory.

Based on the R > 2 criterion, the rheograms presented below (Figure 2, Figure 3, Figure 4, Figure 5, Figure 6 and Figure 7) are all statistically significantly different, even in the case of very close rheograms corresponding to neighboring concentrations.

For example (Figure 2) for ϕ_1_ = 0.66, ϕ_2_ = 0.65 and γ˙ = 1 s^−1^ the values of viscosities are ${\bar{\eta }}_{1}$ = 2.7 Pa·s and ${\bar{\eta }}_{2}$ = 2.3 Pa·s, leading to R = 4. Similarly (Figure 4), for ϕ_1_ = 0.70, ϕ_2_ = 0.68 and ω = 1 rad·s^−1^ the values of the moduli are G′_1_ = 90 Pa and G′_2_ = 80 Pa, leading to R = 3.

## 3. Results and Discussion

### 3.1. General Features of BSA-Stabilized Emulsions

The average droplet size D(4.3) of emulsions with varying oil fraction and constant protein concentration is shown in Figure 1a. The average droplet size varies from 20 μm to 40 μm depending on ϕ. The droplet size slightly increases from ϕ = 0.1 to ϕ = 0.6 and, then, decreases until the highest oil volume fraction. The decrease in D(4.3) is closely related to the jamming transition. Once the transition from a pseudo-liquid to a pseudo-solid fluid takes place, the effective viscosity of the medium increases and provides additional torque during emulsification, leading to a decrease in drop size. This is also confirmed in Figure 1b, where the vanishing of rheological properties is used to determine the critical fraction, at which this transition begins. By comparing both figures we confirm that D(4.3) starts to decrease in that same transition zone. Additionally, another important factor that impacts D(4.3) with ϕ is the behavior of protein as emulsifiers. As any emulsifier, proteins have an optimal concentration (Copt) for achieving optimal emulsifying conditions above and below which droplet size decreases or increases [42]. When ϕ varies_,_ the ratio between available emulsifier and the surface area to be stabilized is altered. Under diluted conditions, the average droplet size is influenced by the combined stabilization mechanisms exhibited by BSA and how they are affected by the changing ratio. This complex process may explain the initial increase in droplet size before the jamming transition. It is also interesting to compare the average droplet sizes after 1 month to those just after preparation (Figure 1a). No difference can be pointed out indicating that all the emulsions remain stable against coalescence regardless of the dispersed-phase fraction under refrigerated conditions (4 °C) for at least a month. This emphasizes that 1 wt.% of BSA stabilize the emulsions with high oil volume fractions.

The determination of ϕ_C_ is a crucial point in the scaling procedure of BSA-stabilized emulsions. This can be achieved by considering the vanishing of G′_p_ and $\sigma_{y}$ with ϕ. Indeed, both properties are closely related to the jamming transition since they become non-zero above ϕ_C_ [15,43]. To determine ϕ_C,_ quadratic fittings were applied for each property and are depicted in Figure 1b. Another alternative for ϕ_C_ determination is also to fit a linear regression law considering the first non-zero values of G′_p_ and σ_y_. The data for σ_y_ and for G′_p_ are extracted from rotational and oscillatory experiments as it will be shown in the following sections. From this, we defined ϕ_C_ = 0.634 ± 0.02, which interestingly remains close to ϕ_RCP_ as for surfactant-stabilized emulsions. The detail on ϕ_C_ determination for each method is given in Table S1 in the Supplementary Materials. Then, by applying Equation (4) on each rheological property, similar values of Δ and B exponents within experimental error (Δ = 1.92 ± 0.17, B = 2.20 ± 0.34) are obtained, remaining close to 2, as reported by other studies [4] and described on the following section.

Another advanced characterization technique was utilized to observe the structural changes in the emulsion at the microscopic level. Fluorescence intensity images of emulsions stabilized at different dispersed-phase oil fractions of 0.3, 0.66, and 0.80 are displayed in Figure 1c. The choice of the dispersed-phase oil fractions of 30%, 60% and 80% is made to highlight 3 different structures of the emulsion. The dispersed phase of 30% concerns the diluted regime, where small amounts of droplets are present. The droplets are far from each other. At a dispersed-phase fraction of 0.66, the droplets are packed in contact all together and densely packed. This confirms that the ϕc is lower than 0.66. This is coherent with the previously determined value of the ϕc of 0.634. In addition, for the dispersed-phase fraction of 80%, the droplets are still in contact but the emulsion follow a transition from densely packed spherical droplets (at ϕ = 0.66) to hexagonally packed droplets (at ϕ = 0.80). The microscopic structure analysis indicates that it is expected that the emulsions will exhibit a liquid-like behavior for the dispersed phase lower than ϕc (<0.63), while a solid-like behavior occurs for dispersed-phase fractions larger than ϕc (>0.63–0.64). It can be also used to verify the relationship between macroscopic rheological behavior and microscopic structure.

### 3.2. Scaling of Flow Behaviour

Shear rate sweeps with varying dispersed-phase volume fractions are presented in Figure 2. In general, for a given shear rate, shear stress increases with the oil fraction over the entire range of ϕ. Below the critical volume fraction, the data present shear-thinning behavior at high shear rates. For data above ϕ_C_, a yield stress was observed at low shear rates. Under high shear rates, data for ϕ > ϕ_C_ also presented shear-thinning behavior. The Herschel–Bulkley model was applied to ϕ > ϕ_C_ curves. In the measurement conditions for rotational tests, it is not possible to access the first viscosity plateau for ϕ < ϕ_C_. Because of this, no reliable fitting of Equation (3) could be evaluated.

The scaling framework application to flow curves of BSA-stabilized emulsions is shown in Figure 3. The scaling was performed for emulsions above ϕ_C_. Shear rate and shear stress data in the supercritical region were mapped as a function of the distance to ϕ_C_. Supercritical curves collapsed into a master curve after adjusting the scaling parameters Γ and Δ to 3.85 and 2.15, respectively. Note that the Δ value remained close to 2 and in the same region as the values obtained when applying Equation (4). The obtained supercritical master curve was accurately fitted to Equation (1) with β = 0.558 and K = 1.73 Pa s^β^. The subcritical data automatically collapsed into a secondary master curve and overlapped in the shear-thinning region at high shear rates. The Cross equation was fitted to the secondary master curve based on the fitting parameters from the supercritical curve. Considering Equation (3), the Cross equation can be rewritten as $\sigma_{scaling}=\eta_{s}\;{\phi_{c}^{m}\dot{\gamma }}_{scaling}\dot{/\left(({{1+(\eta }_{s}\phi }_{c}^{m}/K)\right){\dot{\gamma }}_{scaling}^{\left(1-\beta \right)}})$ [36]. The secondary master curve was correctly fitted with a solvent viscosity η_s_ = 4.5 × 10^−3^ Pa·s. Because there are BSA proteins in the bulk, the value for η_s_ is slightly greater than pure water viscosity. Cross parameters were deduced directly from the Herschel–Bulkley fit of the supercritical branch. When comparing our samples with mobile and rigid emulsions studied by Dinkgreve et al., the scaling parameters Γ and Δ exhibit almost identical values [30]. This suggests that the modelling of the flow behavior of Pickering emulsions stabilized with proteins can be assimilated to that of emulsions with surfactants. The correct use of the scaling approach demonstrates that it is not necessary to modify the modelling technique to describe the flow behavior whatever the nature of the emulsion. In fact, through our results, it can be considered that the universality of this framework can be extended to complex systems of protein-stabilized Pickering emulsions due to the similar scaling parameters. This represents a great opportunity as it can be applied to multiple complex systems at an industrial level.

### 3.3. Extension to Oscillatory Rheology

The oscillatory rheology was used to not destructure and deconstruct the emulsion sample [44,45]. It helps to follow the structure and the structural dynamic in the linearity domain for which the mechanical solicitation is sufficiently low to not affect the structure nor the structural dynamic [45,46,47]. The oscillatory rheology was used to probe the emulsion without perturbation. The oscillatory rheology is then a complementary approach from the previous flow curves since the flow curves significantly affect and perturbate the sample since it produces the flow of the emulsion (due to van der Waals link breakage or droplet deformation, etc.).

Frequency sweeps presenting $\left|G^{*}\right|$ modulus variation with ϕ for BSA-stabilized emulsions are shown in Figure 4. $\left|G^{*}\right|$ as a function of ϕ follows the same trend as the shear stress in rotational measurements, presenting a monotonic increase with ϕ. Above the jamming fraction, $\left|G^{*}\right|$ tends to a plateau behavior. Under these conditions, the rheology is mainly that of a highly elastic matrix of packed droplets. Below the critical fraction, however, $\left|G^{*}\right|$ curves tend towards the same values in the high frequency region (above 10 rad/s). To gain a better understanding, a closer look of the storage and loss moduli is given in Figure 5a for two samples below and above ϕ_C_. For supercritical conditions, a predominant and plateau behavior of G′ over G″ is observed supporting the solid-like nature of samples. The viscoelastic behavior differs, however, below the jamming fraction as G′ and G″ overlap as for soft gels. This aspect may suggest a strong influence of the bulk phase in the rheology of diluted emulsions. To explore such effect, the protein aqueous solution is also characterized as presented in Figure 5b. Modulus $\left|G^{*}\right|$ is higher for diluted emulsions than for solution. As the frequency increases, the gap between $\left|G^{*}\right|$ of solution and emulsions narrows to overlap in the region above 10 rad/s as observed before. Since measurements are performed in the linear domain, these results confirm that the dilute regime behavior for oscillatory rheology is more due to a contribution of the BSA matrix in the continuous phase than to the presence of oil droplets.

Subsequently, the scaling of oscillatory rheological behavior is applied and shown in Figure 6a. $\left|G^{*}\right|$ and ω were treated analogously as σ and $\dot{\gamma }$ so that: $\omega_{scaling}=\omega /{\left|\Delta \phi \right|}^{\Gamma }$ and $G_{scaling}^{*}=G^{*}/{\left|{\Delta \phi }_{J}\right|}^{\Delta }$. Ideally, a relationship between rotational and oscillatory rheology would be coupled by an equivalence of the scaling parameters for both cases. Therefore, no modification of Γ and Δ used in rotational tests was performed (Γ = 3.85 and Δ = 2.15). As for flow curves and simulation works, supercritical oscillatory data tend to collapse into a master curve [4,38]. Results show that the supercritical collapsed data in the oscillatory regime behaves similarly as the scaled flow curves. However, the subcritical data did not collapse as for rotational measurements. Due to this, no proper fitting of Equation (2) was obtained.

To analyze this discrepancy, three different approaches were adopted. First, an equivalent form of the Herschel–Bulkley model was fitted to the supercritical master curve as: $G^{*}=G_{1}+K_{G}{\dot{\gamma }}^{\beta_{G}}$. The resulting fit provided G_1_ = 34,532.1, K_G_ = 67.94 Pa.s^βG^ and β_G_ = 0.435. An overlap of rotational and oscillatory scaling would also imply equivalent values for the parameters β and β_G_. However, this is not the case here. Secondly, the scaling of oscillatory data was also assessed using the Herschel–Bulkley fit produced by the scaling of flow curves. For this purpose, the obtained shear stress data from the fit of Figure 3 was displaced as: G* = σ_FlowCurves_/γ_y_, where γ_y_ = 0.07 is the yield strain used. The fitting of the Cross model from flow curves was treated in a similar way with γ_y_ = 0.07. The value of γ_y_ was verified through a graphical tangent method from strain sweeps (see Figure S3 of the Supplementary Materials). From both fits, it is possible to observe the difference between rotational and oscillatory results when applying the scaling framework.

Although we find a master curve in the supercritical region, the difference between fit parameters shows that further modifications are necessary in order to describe the oscillatory behavior through rotational behavior. Finally, another way to identify equivalences between these two behaviors is to verify if the Cox–Merz rule (CMR) applies to our BSA-stabilized emulsions. This empirical rule is widely used in polymeric and colloidal systems and states that the steady shear viscosity and the complex viscosity have the same values for $\dot{\gamma }$ = ω [48]. While the CMR has been widely used, the reasons why it applies correctly in some cases, but not always, is still an ongoing work. For comparison purposes, the CMR can be rewritten in terms of shear stress and $\left|G^{*}\right|$ modulus as proposed by Winter [49]:

(7) $$CMR:\eta \left(\dot{\gamma }\right)=\eta^{*}\left(\omega \right)\;\to \;\sigma \left(\dot{\gamma }\right)=\left|G^{*}\left(\omega \right)\right|$$

Cox–Merz rule was applied to oscillatory data of BSA-stabilized emulsions as presented in Figure 6b for some samples (see Figure S4 in the Supplementary Materials for the classical CMR application). No good agreement between rotational and oscillatory results is observed as G*(ω) data do not overlap with σ($\dot{\gamma }$) data. This was also identified in literature for BSA solutions presenting η* data above η [50]. In Pickering emulsions stabilized with BSA particles, the approaches tested demonstrate the challenge of characterizing oscillatory rheology through rotational rheology. For this type of systems, such an equivalence is not suitable.

In a similar manner to the scaling in Figure 5, an overlap can be achieved by using a constant yield strain of 0.07 as presented in the inset of Figure 6b. This is because the rate between G modulus and σ_y_ remains constant also indicating that the extent of inter-particle interactions remains constant. However, to explain the observed discrepancy, we must consider the structural changes that occur in the colloidal system after surpassing the yield stress in rotational tests. Such changes do not occur in oscillatory measurements due to the limited deformations applied, which keep the samples within the linear viscoelastic regime. Although it is known that emulsions do not exhibit thixotropy (see Figure S2 of the Supplementary Materials), it is necessary to consider modifications related to the conformation of BSA and the overall fluid structure. The interactions between BSA particles at the interfaces of oil droplets and in the bulk, along with the resulting rheological behavior stemming from these interactions, introduce significant variability between oscillatory and rotational measurements. Indeed, previous studies on Pickering emulsions with silica nanoparticles have shown that complex bulk phases strongly influence the rheological behavior [15]. Thus, with varying rheological measurements, the bulk phase impacts the overall rheological behavior differently. On the other hand, it is worth noting that BSA interactions come mainly from their non-isotropic charge distribution due to their macro-molecular nature [39,40,51,52]. This implies that even in isoelectric conditions it is expected that slight inter-particle electrostatic interactions occur. Under so many sources of uncertainty, it is hardly normal that it is not possible to describe oscillatory behavior through flow behavior. Nevertheless, the collapse of the supercritical section demonstrates that this framework may be successfully applied to other simpler systems.

To conclude, it seems interesting to summarize graphically the models to give a more intuitive understanding (Figure 7).

## 4. Conclusions

The rheological behavior of silicone oil-in-water Pickering emulsions stabilized with bovine serum albumin soft particles was studied in this article. The flow behavior of emulsions stabilized with proteins was successfully described using a phase transition analogy, based on the jamming fraction and two scaling parameters. The values of the scaling parameters used for BSA-stabilized emulsions remained remarkably similar to those observed for classical surfactant-stabilized emulsions from previous works. Such similarity suggests that, from a rheological point of view, Pickering emulsions stabilized with globular proteins can be described in the same manner as surfactant-stabilized emulsions. Additionally, the results on BSA-stabilized emulsions provide further evidence that the scaling parameters Δ and Γ may be universal or, at least, belong to some kind of universality for athermal systems.

Moreover, the viscoelastic behavior of emulsions was studied through oscillatory rheology. An attempt was made to extend the scaling of flow curves to the oscillatory regime. In the first instance, the rescaling of oscillatory data appear to collapse the curves in the same manner as in rotational tests. However, several verifications, including non-compliance with the Cox–Merz rule, demonstrate the impossibility of extending this methodology to protein-stabilized emulsions. The possibility of correcting the rescaling through the yield strain is a promising option, and its study is part of the future work to be performed on this framework. Further work on the extension of this modeling framework to oscillatory rheology also includes tests on systems following the Cox–Merz rule, such as polymer solutions.

Concerning the practical benefit of this study, it appears that we could have universal behavior whatever the type of emulsifier. As seems to be the case, rescaling can be performed with other types of proteins such as vegetal proteins. This will allow us to better understand whether the behavior of proteins as emulsion stabilizers is closer to particles or traditional surfactants. Understanding this behavior is important in order to develop more efficient and customizable emulsion systems specifically tailored to specific industrial applications including controlled drug release or improved sensory attributes in food products, presents exciting possibilities.

## Data Availability

The data that underlie the results that are presented in this paper are not publicly available at this time but can be obtained from the authors upon reasonable request.

## Supplementary Materials

The following supporting information can be downloaded at: https://www.mdpi.com/article/10.3390/nano15090650/s1, Figure S1. Modeling the rapid increase in G′_P_ in silicone oil-in-water Pickering emulsions stabilized with 1 wt% BSA particles as a function of the dispersed-phase volume fraction ϕ. The blue line represents the fitting of the Mougel model, the blue dotted line corresponds to the fitting of Paruta’s equation and the green dotted line is the Princen–Kiss fitting. Figure S2. Thixotropy verification on an oil-in-water emulsion with Φ = 0.80 and 1 wt% BSA. Stress relaxation tests are carried out in increasing and decreasing directions from low to high shear rates (upward triangles) and from high to low shear rates (downward triangles). Figure S3. Strain sweeps of high dispersed-phase oil fraction emulsions stabilized with BSA and of a BSA solution (upward triangles). The yield strain γ_y_ (red circle) is determined with a tangent method based on the G′p value and the tangent with the non-linear region. Figure S4. Cox–Merz rule applied to viscosity and complex viscosity of silicone oil-in-water emulsion with ϕ > ϕ_C_ and 1 wt% BSA. Table S1. Data resulting from fitting quadratic and linear equations to σ_y_ and G′p in Figure 1b, used to estimate the $\phi_{c}$ value in emulsions stabilized with 1 wt% BSA. References [53,54,55,56] are cited in the supplementary materials.
