# Supplementary material for: Rescaling Flow Curves of Protein-Stabilized Emulsions
Source: Nanomaterials (Basel). 2025 Apr 25;15(9):650. doi: 10.3390/nano15090650 (PMC12073574; doi:10.3390/nano15090650)
Supplement: Supplementary file 1 [file nanomaterials-15-00650-s001.zip › nanomaterials-3517736-supplementary-new.pdf]

nanomaterials

Supplementary Materials

Rescaling Flow Curves of Protein-Stabilized Emulsions

Santiago F. Velandia [1,2], Philippe Marchal [1], Véronique Sadtler [1], Cécile Lemaitre [1], Daniel Bonn [2] and Thibault Roques-Carmes [1,*]

[1] Université de Lorraine, CNRS, LRGP, F-54000 Nancy, France; sf.velandia10@gmail.com (S.F.V.); philippe.marchal@univ-lorraine.fr (P.M.); veronique.sadtler@univ-lorraine.fr (V.S.); cecile.lemaitre@univ-lorraine.fr (C.L.)
[2] Van der Waals-Zeeman Institute, Institute of Physics, University of Amsterdam, Science Park 904, 1098 XH Amsterdam, The Netherlands; d.bonn@uva.nl
* Correspondence: thibault.roques-carmes@univ-lorraine.fr

S1. Modeling G modulus

Three rheological models besides the power law presented in the main text are applied to describe the G'p behavior. These are shown in Figure S1. Paruta-Tuarez et al. (equation S1) [1], Princen/Kiss (equation S2) and Mougel (equation S3) models are introduced here:

$$G = \varepsilon \, \Sigma \, \Delta\phi^{t} \quad \text{(S1)}$$

As mentioned in the main text, equation S1 is a power law-based model containing the Laplace pressure Σ as a stress scale so that: $G_0 = \varepsilon \, \Sigma^{t}$. For BSA-stabilized emulsions we considered R = 20 μm and γ = 12 mN/m while ε and t are adjustable parameters. The results obtained from this fit are equivalent as for equation S4 in the main text. On a similar note, Princen and Kiss model (equation S2) also considers Σ and is a simplified form of equation 4 in the main text, usually applied to emulsions with surfactants.

$$G' = \alpha \, \Sigma \, \phi^{\frac{1}{3}}(\phi - \Phi_c) \quad \text{(S2)}$$

Mougel model, however, is more robust than Princen and Kiss equation since it considers an intermolecular distance parameter D_0 between droplets to account for jamming effects [2].

$$G' = \frac{2\,A\,\pi\,D0\,\phi\,\dot{\gamma}}{\dot{R}^2\,(\Phi_{MAX} - \phi)} \quad \text{(S3)}$$

The latter includes A as an adjustable parameter, again R as the average drop radius, Φ_{MAX} is the maximum volume fraction that can be dispersed and γ the interfacial tension. In our case, these values were taken equivalently as for equation S1 while A^*D_0 and Φ_{MAX} were considered as adjustable parameters. Results showed that Φ_{MAX} = 0.88 and A^*D_0 = 8.9 10^{-7} with an R^2 = 0.95. In the same vein, the Princen model provided: α = 8.5 and ϕ_c = 0.66 with R^2 = 0.92.

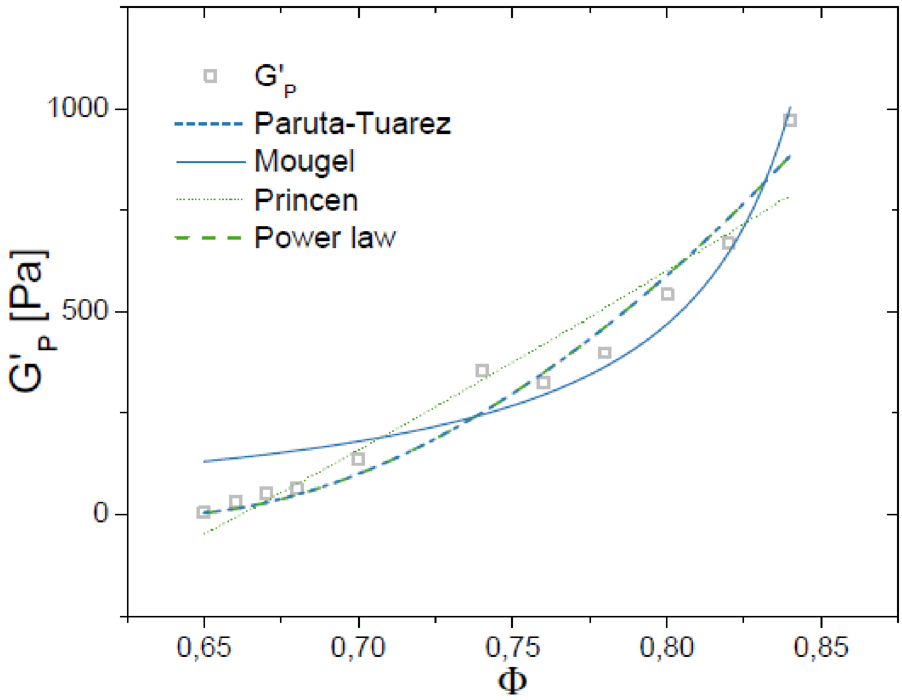

Figure S1. Modeling the rapid increase of G'p in silicone-oil-in-water Pickering emulsions stabilized with BSA particles.

Interestingly, both Mougel and Princen/Kiss models do not correctly describe the behavior of the samples above the jamming transition. It has been shown before that Princen and Kiss equation tend to underestimate the rapid increase of G'p in surfactant-stabilized emulsions [3]. In the case of Pickering emulsions it has also been shown that such increase also exist and tend to show a rapid progression compared to emulsions with surfactants [4]. Thus, the inability of this equation to correctly describe the behavior is understandable. Concerning Mougel equation, we observe a similar case. This fitting considers better the rapid increase of G'p at high oil volume fraction but lacks to properly describe the initial increase of G'p with close to the jamming fraction ϕ_c. The reason, again, is related to the rapid increase for our BSA-stabilized Pickering emulsions, especially after $\phi = 0.80$. As seen in the main text, the power law approximation (that follows the same type of equation and results as Paruta-Tuarez model) remains the best to describe this behavior as well as determining ϕ_c.

S2. Thixotropy verification

Stress relaxation tests where done in order to verify if samples presented thixotropy and are shown in Fig. S2. For that matter, shear rate intervals (0.1 s^{-1}, 1 s^{-1}, 10 s^{-1} and 100 s^{-1}), each with a duration of 100 s, were applied in increasing and decreasing directions to an emulsion sample at high dispersed phase fraction. In any evaluated shear rate, the steady state is reached during the first seconds. The shear stress values overlap in both directions of the measurements, showing that these samples do not present thixotropy.

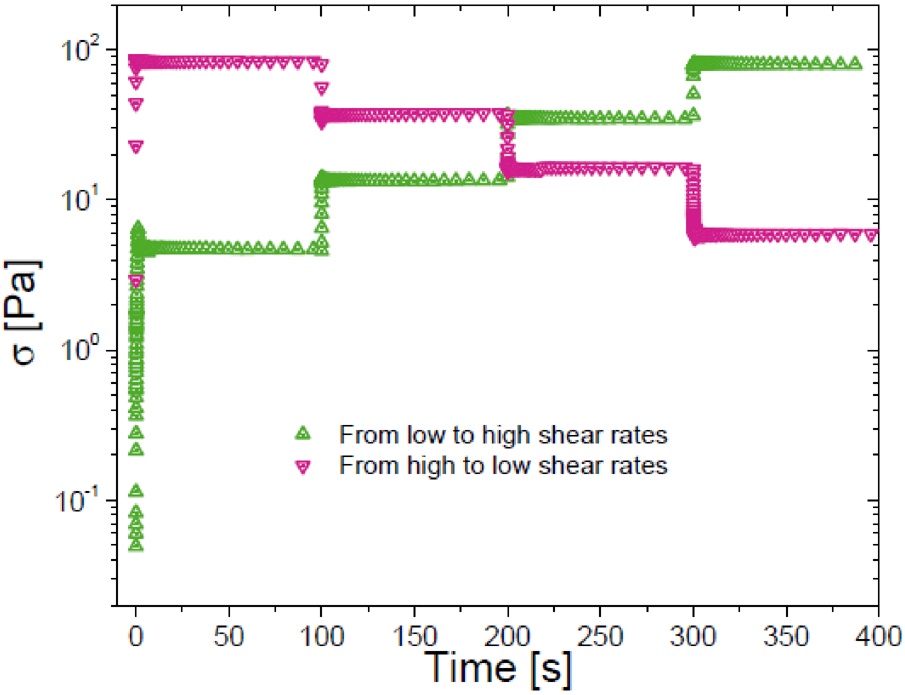

Figure S2. Thixotropy verification on an oil-in-water emulsion with = 0.80 and 1%wt BSA. Stress relaxation tests are carried out in increasing and decreasing directions from low to high shear rates (Upward triangles) and from high to low shear rates (Downward triangles).

S3. Yield stress determination

The tangent method to determine the yield strain from strain sweeps is depicted in Fig.S3. This method was also used for Pickering emulsions stabilized with silica nanoparticles in the literature [5].

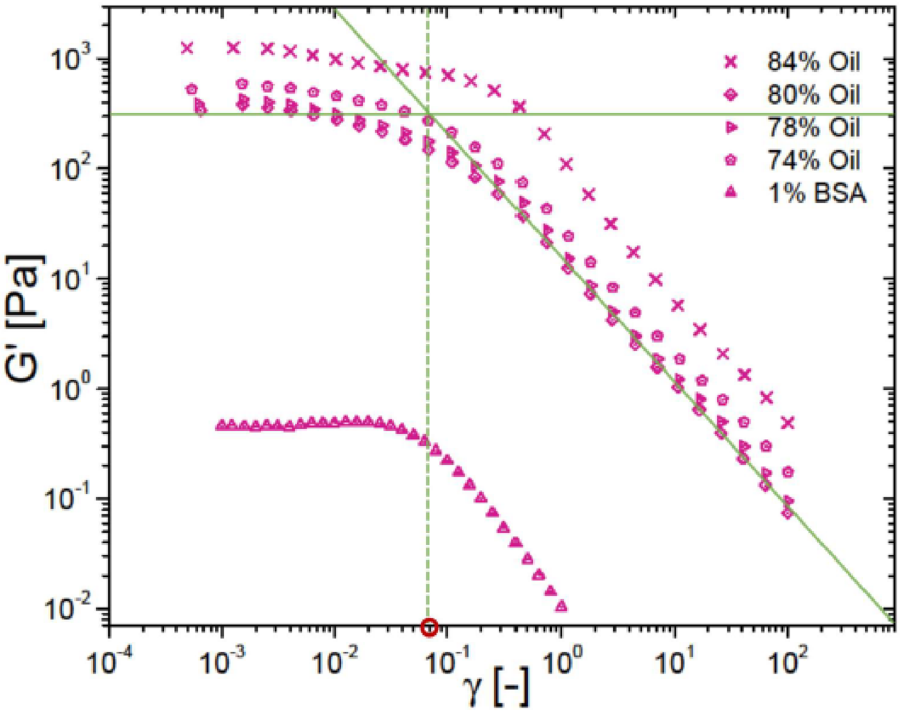

Figure S3. Strain sweeps of high dispersed phase oil fraction emulsions stabilized with BSA and of a BSA solution (upward triangles). The yield strain γ_y (red circle) is determined with a tangent method based on the G′p value and the tangent with the non-linear region.

S4. Cox-Merz rule

The classical Cox-Merz rule applied to shear viscosity and complex viscosity is presented in Fig.S4.

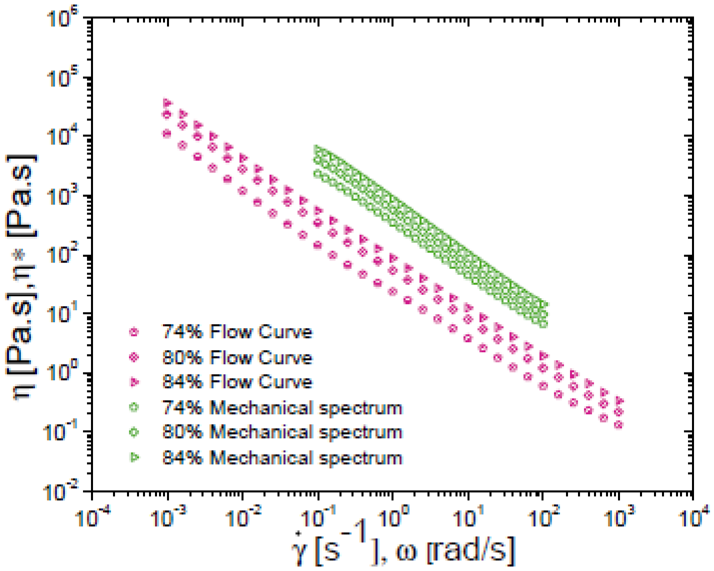

Figure S4. Cox Merz rule applied to viscosity and complex viscosity of silicone oil-in-water emulsion with $\phi > \phi_c$ and 1%wt BSA.

As in the main text, oscillatory and rotational data do not overlap.

S5. Jamming fraction determination

The data for each fitting performed on the rheological properties G′p and σ_y of Figure 1b are shown in Table S1. The quadratic fitting followed $\sigma_y = \alpha\,(\phi - \phi_c)^2$ or $G'_p = \alpha\,(\phi - \phi_c)^2$ with α and ϕ_c fitting parameters whereas the linear approximation followed $\sigma_y = g\,\phi + d$ or $G'_p = g\,\phi + d$ with g and d also fitting parameters.

Table S1. Data resulting from fitting quadratic and linear equations to G′p and σ_y in Figure 1b, used to estimate ϕ_c value in emulsions stabilized with 1 wt% BSA.

Property	Fitting	ϕ_c	Error	α parameter	b parameter
σ_y	Quadratic	0.591	0.014	387.5	1.92 ± 0.169
σ_y	Linear	0.648	0.035	-	-
G′p	Quadratic	0.634	0.020	33630.7	2.21 ± 0.337
G′p	Linear	0.645	0.091	-	-

In general, it is observed that ϕ_c tends to ϕ_{RCP} for three out of four fitting cases, except for the quadratic equation applied to σ_y in rotational tests, where a lower value of 0.591 is obtained for the critical condition. As we aim to apply the scaling methodology to protein-stabilized emulsion data, following the same procedure as in other studies is ideal. Generally, the quadratic approximation has been preferred in the past by other authors. In both cases of quadratic fitting, relatively low errors are obtained considering that our samples are polydisperse. However, the values of σ_y are deduced from the fitting of the HB equation, while G′p is obtained by averaging the plateau values in oscillatory tests. The estimation of these parameters in protein emulsions appears to be more affected in rotational tests than in oscillatory tests due to the bulk rich in proteins, as mentioned in the main text (see Results and Discussion section). Therefore, in this study, we use the value of ϕ_c = 0.634 obtained from the quadratic approximation to the G′p data.

References

1. E. Paruta-Tuarez and P. Marchal, "Association of Percolation Theory with Princen's Approach To Model the Storage Modulus of Highly Concentrated Emulsions," Industrial and engineering chemistry research, vol. 52, no. (33), pp. 11787–11791, 2013.
2. J. Mougel, O. Alvarez, C. Baravian, F. Caton, P. Marchal, M.-J. Stébé, and L. Choplin, "Aging of an unstable w/o gel emulsion with a nonionic surfactant," Rheol Acta, vol. 45, pp. 555–560, June 2006.
3. E. Paruta-Tuarez, P. Marchal, V. Sadtler, and L. Choplin, "Analysis of the Princen and Kiss Equation To Model the Storage Modulus of Highly Concentrated Emulsions," Industrial & Engineering Chemistry Research, vol. 50, pp. 10359–10365, Sept. 2011.
4. S. F. Velandia, P. Marchal, C. Lemaitre, V. Sadtler, and T. Roques-Carmes, "Evaluation of the repartition of the particles in Pickering emulsions in relation with their rheological properties," Journal of Colloid and Interface Science, vol. 589, pp. 286–297, May 2021.
5. S. F. Velandia, D. Ramos, M. Lebrun, P. Marchal, C. Lemaitre, V. Sadtler, and T. Roques-Carmes, "Exploring the link between interfacial and bulk viscoelasticity in reverse Pickering emulsions," Colloids and Surfaces A: Physicochemical and Engineering Aspects, vol. 624, p. 126785, Sept. 2021.

Disclaimer/Publisher's Note: The statements, opinions and data contained in all publications are solely those of the individual author(s) and contributor(s) and not of MDPI and/or the editor(s). MDPI and/or the editor(s) disclaim responsibility for any injury to people or property resulting from any ideas, methods, instructions or products referred to in the content.
